# Seasonal and Diel Activity Patterns of Eight Sympatric Mammals in Northern Japan Revealed by an Intensive Camera-Trap Survey

**DOI:** 10.1371/journal.pone.0163602

**Published:** 2016-10-12

**Authors:** Takashi Ikeda, Kenta Uchida, Yukiko Matsuura, Hiroshi Takahashi, Tsuyoshi Yoshida, Koichi Kaji, Itsuro Koizumi

**Affiliations:** 1 Faculty of Environmental Earth Science, Hokkaido University, Sapporo, Hokkaido, Japan; 2 Graduate School of Environmental Earth Science, Hokkaido University, Sapporo, Hokkaido, Japan; 3 Hokkaido Research Center, Forestry and Forest Products Research Institute, Sapporo, Hokkaido, Japan; 4 Kansai Research Center, Forestry and Forest Products Research Institute, Kyoto, Kyoto, Japan; 5 Wildlife Management Laboratory, Rakuno Gakuen University, Ebetsu, Hokkaido, Japan; 6 Laboratory of Wildlife Management, Tokyo University of Agriculture and Technology, Fuchu, Tokyo, Japan; University of Texas Southwestern Medical Center, UNITED STATES

## Abstract

The activity patterns of mammals are generally categorized as nocturnal, diurnal, crepuscular (active at twilight), and cathemeral (active throughout the day). These patterns are highly variable across regions and seasons even within the same species. However, quantitative data is still lacking, particularly for sympatric species. We monitored the seasonal and diel activity patterns of terrestrial mammals in Hokkaido, Japan. Through an intensive camera-trap survey a total of 13,279 capture events were recorded from eight mammals over 20,344 camera-trap days, i.e., two years. Diel activity patterns were clearly divided into four categories: diurnal (Eurasian red squirrels), nocturnal (raccoon dogs and raccoons), crepuscular (sika deer and mountain hares), and cathemeral (Japanese martens, red foxes, and brown bears). Some crepuscular and cathemeral mammals shifted activity peaks across seasons. Particularly, sika deer changed peaks from twilight during spring–autumn to day-time in winter, possibly because of thermal constraints. Japanese martens were cathemeral during winter–summer, but nocturnal in autumn. We found no clear indication of predator-prey and competitive interactions, suggesting that animal densities are not very high or temporal niche partitioning is absent among the target species. This long-term camera-trap survey was highly cost-effective and provided one of the most detailed seasonal and diel activity patterns in multiple sympatric mammals under natural conditions.

## Introduction

Day and night cycles, although highly variable across latitudes and seasons, are ubiquitous on our planet, which imposes a near universal selection pressure on most organisms. The development of a 24hr circadian rhythm is one of the most notable outcomes. Diel time partitioning should be structured among different sympatric species, particularly those under predator-prey and competitive interactions.

In general, terrestrial mammalian activity patterns can be categorized into diurnal, nocturnal, crepuscular (active at twilight), and cathemeral (active throughout the day) [1]. However, activity patterns are highly variable among regions and across seasons, even within the same species. Many factors can affect activity patterns, such as day length [1], temperature [1], precipitation [2], predator-prey or competitive interactions [3,4,5], and human activities [6]. Additionally, some studies reported that diel activity patterns were influenced by the physical characteristics of each species, such as visual systems [7] and the relative size of corneal and transverse eye diameters [8]. Such complex interactions obscure our understanding of the underlying processes governing activity patterns and selection pressures. In particular, we are lacking basic quantitative data on the details of activity patterns in sympatric mammal species. If two potentially interacting species such as predator and their preys or competitors changing their diel activity in separate ways [9], the role of temporal niche partitioning may be inferred. Furthermore, if only one species changes activity patterns, other factors such as temperature constraints or a diet shift may be suggested as temporal habitat use. Such information would also be useful for conservation and management programs of rare, invasive, or overabundant species.

Mammalian activity patterns have been evaluated mainly through direct observation [10] or telemetry surveys [11], requiring high survey and capture efforts. In addition, disturbances caused by direct observation and live capture can bias the results or negatively affect animal populations. To overcome these challenges, camera-trap survey has received increasing attention during the last decade. This survey method has been used to monitor the activity patterns of some mammal species in detail [12,13,14,15]. However, most studies only monitored over relatively short-term periods (e.g. a single season), and a lack of data on seasonal and diel activity patterns makes it difficult to come to any conclusions. Moreover, they were unable to discern any diel activity patterns by camera-trap survey.

Here we conducted an intensive long-term camera-trap survey in boreal forest of northern Japan, collecting data sets on eight mammals with different body sizes, ranging from Eurasian red squirrels (*Sciurus vulgaris*) to brown bears (*Ursus arctos*), and were able to clearly define diel activity pattern. We aimed to evaluate the seasonal and diel activity patterns of sympatric mammals, and also discuss potential interspecific interactions. This data set provides not only the basic biological data for each species but also significant insights into temporal niche partitioning.

## Materials and Methods

### Study area

**Fig 1:**
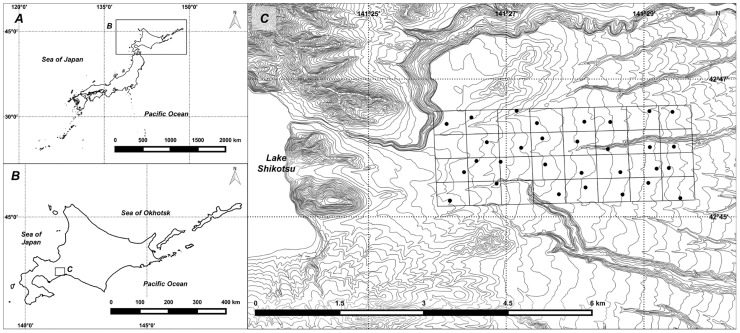
Map of the eastern area on Lake Shikotsu (C) in Hokkaido (B), Japan showing 30 camera-trap sites. We divided the study area into 32 grid cells each 31.25-ha in size and placed infrared-triggered cameras in 30 grid cells (black circles). We made this map ourselves, using contour lines and coastline from Geospatial Information Authority of Japan.

The study area was a relatively pristine habitat of boreal forest near Lake Shikotsu, Hokkaido, Japan (1,000 ha, 42° 45′ N, 141° 27′ E; Fig 1). This area was contiguous to Shikotsu-Tōya National Park and hunting has been partially prohibited. The mean annual precipitation is 1,900 mm, and the mean annual temperature is 6.9°C (monthly mean of -6.1°C in January and 20.6°C in August). 12 species of Chiroptera, nine species of Carnivora, eight species of Rodentia, four species of Soricomorpha, one species of Lagomorpha, and one species of Artiodactyla exist in this study area [16]. The dominant vegetation includes coniferous plantations (35.4%), open land (26.8%), mixed forests of conifers and broad-leaved trees (20.4%), and deciduous broad-leaved trees (17.2%). In the coniferous forest, the major canopy species are Sakhalin spruce (*Peces glehnii*), Yezo spruce (*Picea jezoensis*), and Todo fir (*Abies sachalinensis*). In the deciduous forest, the major canopy species are oak (*Quercus crispula*), painted maple (*Acer mono*), and Japanese white birch (*Betula platyphylla*).

### Camera-trap survey

This camera-trap survey in Shikotsu-Tōya national park was conducted with the permission of Hokkaido regional environment office of the Japan Ministry of the Environment, and the permission of the Ishikari and Iburi Tobu District Forest Office of the Japanese National Forest. To investigate the activity patterns of mammals, we used 30 infrared-triggered cameras (LTL Acorn 5210a; LTL Acorn Outdoors, Green Bay, Wisconsin, USA) during June 2012–June 2014. We divided the study area into 32 grid cells each 31.25-ha in size and placed cameras in 30 grid cells (Fig 1C), although we did not set cameras in two grid cells for technical reasons. The camera-trap survey was originally designed to monitor sika deer (*Cervus nippon*); therefore, cameras were placed at animal-trails and dirt roads, where signs of deer were evident. We avoided the sites where frequent human visits were expected. The number of photos is significantly influenced by the difference in animal body size and habitat use, including home range size and territorial behavior that may increase double counting [17,18]. For population density estimates using camera-trap survey, ignoring these problems could result in overestimation or underestimation. This study, however, focused on diel activity pattern rather than population density. Thus, double counting, if it occurred, would not significantly bias the results of the study. To obtain accurate photographed time, we programed a high frequency setting with a 5-min delay between consecutive events and captured three photos per each event. Additionally, we believe that these camera settings minimized the influence of animal size on camera activation and activity pattern, because cameras were set toward animal-trails and dirt roads and strapped to trees approximately one meter above the ground. We conducted camera maintenance (e.g. data extraction data and exchange of cameras when required) once a month and no major technical difficulties were encountered; we believe that the cameras could have functioned for an even longer period, possibly up to six months.

All photos taken by the cameras recorded the date and time. To clarify the seasonal and diel activity patterns of mammals, we defined seasons as winter (January–March), spring (April–June), summer (July–September), and autumn (October–December). The average day-lengths and mean daily temperature was 10 h 38 min (9 h 06 min–12 h 40 min) and -4.4°C (-10.2–6.5°C) in winter, 14 h 25 min (12 h 43 min–15 h 21 min) and 10.2°C (-0.5–20.4°C) in spring, 13 h 50 min (11 h 48 min–15 h 17 min) and 19.1°C (10.4–24.2°C) in summer, and 9 h 59 min (9 h 02 min–11 h 45 min) and 4.1°C (-10.5–16.4°C) in autumn. We classified the recorded time of each photo into three time periods; day-time (from 1hr after sunrise to 1hr before sunset), night-time (from 1hr after sunset to 1hr before sunrise), and twilight (1hr before and after sunrise and sunset), according to previous studies (e.g. [19]).

### Data analysis

To estimate the seasonal and diel activity patterns of mammals, we used the kernel density analysis, which is a non-parametric method for evaluating the probability density function of a random variable [20,21]. To determine four diel activity patterns (i.e., diurnal, nocturnal, crepuscular, and cathemeral), we calculated photographic frequencies (the number of photos per hour) per 100 trap-days for three time periods in each season. We defined crepuscular behavior as having photo events more frequently during twilight, diurnal as having photo events more frequently during day-time, and nocturnal as having photo events more frequently during night-time. Cathemeral was defined when no differences were observed in the photographic frequencies among the three time periods. We used one-way ANOVA to compare daily photographic frequencies among the three time periods and the Steel-Dwass multiple comparison test to rank these periods.

To investigate the potential influence of predators on prey species, we used generalized linear mixed models with a Poisson distribution. Japanese martens (*Martes melampus*) and red foxes (*Vulpes vulpes*) are regarded as predators of Eurasian red squirrels and mountain hares (*Lepus timidus*) based on a previous study [16]. We set the total number of prey species (Eurasian red squirrels or mountain hares) photographed as the response variable and the total number of predator species (Japanese martens and red foxes) as the explanatory variables. Each camera site was included as a random intercept. The positive values indicate that prey species (Eurasian red squirrels or mountain hares) may be predated upon by predator species (Japanese martens and red foxes). Similarly, we investigated competitive interactions using a similar model. After the introduction of invasive raccoons (*Procyon lotor*), resource competition among red foxes, raccoon dogs (*Nyctereutes procyonoides*), and raccoons was suggested, resulting in red foxes and raccoon dogs having disappeared in some regions [22]. We set the total number of raccoons photographed as the response variable and the total number of raccoon dogs and red foxes photographed as the explanatory variables. Similar to the model of predator-prey interactions, each camera site was included as a random intercept. The positive values indicate that competitor species (raccoons and raccoon dogs or red foxes) had a similar niche. These analyses were performed in R version 3.1.1 [23] using the glmmML package (Available: https://CRAN.R-project.org/package=glmmML, Accessed 25 April 2016) for the predator-prey and competitive interactions.

## Results

**Fig 2:**
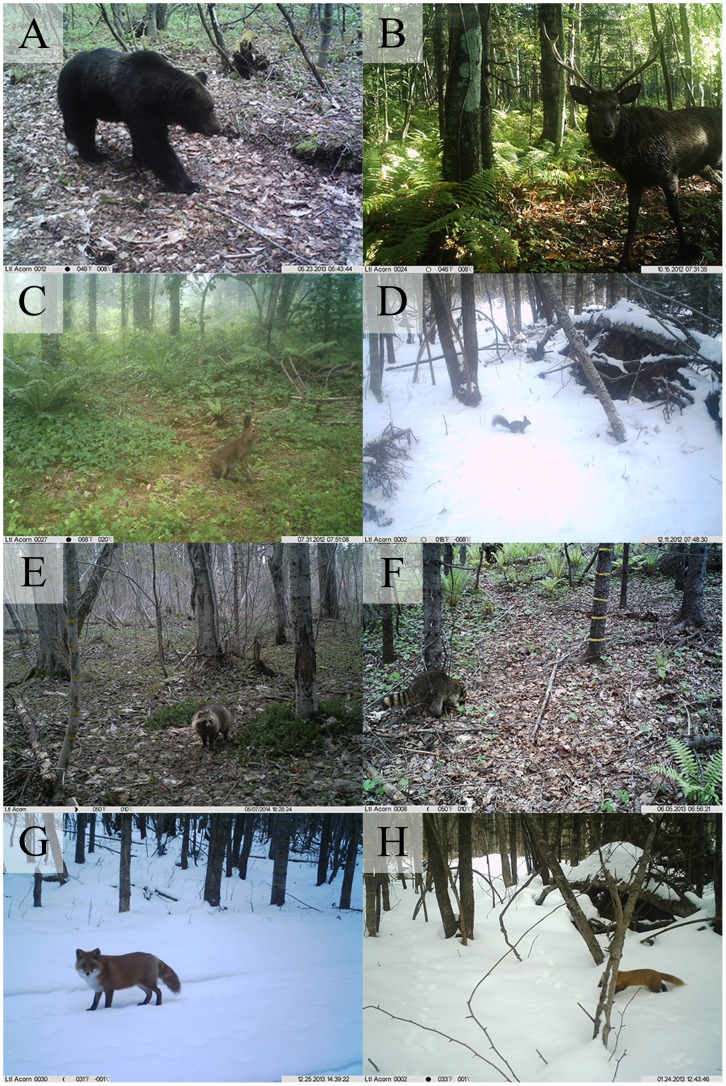
Target mammals taken by infrared-cameras. A, *Ursus arctos*, brown bear; B, *Cervus nippon*, sika deer; C, *Lepus timidus*, mountain hare; D, *Sciurus vulgaris orientis*, Eurasian red squirrel; E, *Nyctereutes procyonoides*, raccoon dog; F, *Procyon lotor*, raccoon; G, *Vulpes vulpes*, red fox; H, *Martes melampus*, Japanese marten. All photos were taken by T. Ikeda.

From 20,344 camera-trap days during June 2012–June 2014, a total of 13,279 capture events were recorded (0.65 photos per camera trap-day on average). Of 35 mammals inhabiting this study area, eight mammals were detected by the infrared cameras (Fig 2). Although flying mammals (e.g. bats and flying squirrels) and small rodents (voles and mice) were not detected, the camera-trap survey succeeded in collecting a large data set for eight mammals ranging in size from Eurasian red squirrels to brown bears (Fig 2). Most of the records were sika deer (84.08%), but more than 60 photos were recorded for other species (mean: 1,660 photos per species, range: 63–11,165, Table 1). Together with the diel activity, seasonal changes in total activity were also revealed from the photos taken. No photos of brown bears were taken during winter, consistent with wintering or hibernation behavior. Activity levels were also significantly reduced during winter in most of the species, except for mountain hares in which the number of photos was highly consistent across seasons.

**Table 1:** Number of photos in each season and percentage of camera-trap photographic records of eight mammals recorded around Lake Shikotsu.

Species	N					Percentage of records (%)
	Winter	Spring	Summer	Autumn	Total	
Squirrel	7	57	110	95	269	2.03
Raccoon dog	18	185	400	129	732	5.51
Raccoon	0	26	31	22	79	0.59
Deer	59	4,200	4,236	2,670	11,165	84.08
Hare	64	71	51	66	252	1.90
Bear	0	28	19	16	63	0.47
Fox	35	222	173	155	585	4.41
Marten	11	34	51	38	134	1.01
Total	194	4,823	5,071	3,191	13,279	100

**Fig 3:**
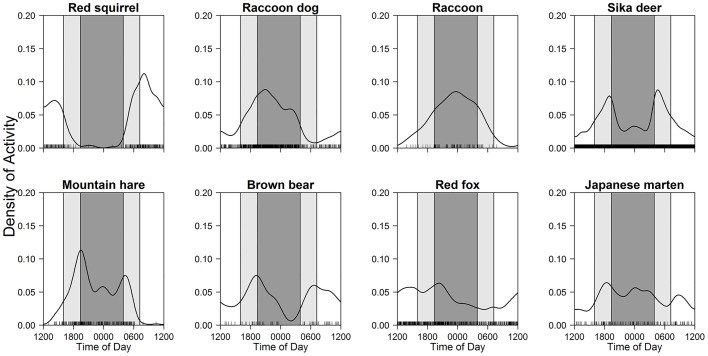
Diel activity patterns of eight mammals throughout the year. Black line and bars, dark grey shaded area, and light grey shaded areas indicate kernel density estimates, daily photo events, night-time, and twilight, respectively.

Comparing average photographic frequencies per 100 trap-days for each mammal among three time periods (one-way ANOVA and Steel-Dwass multiple comparison test) throughout the study period, the diel activity patterns of the eight mammal species were categorized into four patterns (Table 2; Fig 3); diurnal (Eurasian red squirrels), nocturnal (raccoon dogs and raccoons), crepuscular (sika deer and mountain hares), and cathemeral (brown bears, red foxes, and Japanese martens).

**Table 2:** Diel activity patterns throughout the year for each mammal.

Species	Twilight	Day-time	Night-time	Category	*P*
Squirrel	1.28 ± 0.22	2.91 ± 0.22	0.10 ± 0.04	D	***
Raccoon dog	3.86 ± 0.41	1.95 ± 0.18	7.09 ± 0.47	N	***
Raccoon	0.70 ± 0.16	0.10 ± 0.03	0.74 ± 0.12	N	***
Deer	133.26 ± 5.42	50.70 ± 2.07	45.37 ± 2.07	Cr	***
Hare	2.65 ± 0.36	0.31 ± 0.07	1.86 ± 0.18	Cr	***
Bear	0.44 ± 0.13	0.35 ± 0.07	0.24 ± 0.06	Ca	0.32
Fox	2.75 ± 0.31	3.25 ± 0.23	3.65 ± 0.27	Ca	0.06
Marten	0.67 ± 0.15	0.56 ± 0.08	0.91 ± 0.12	Ca	0.11

These values indicate average photographic frequencies and *SE* per 100 trap-days. Differences in the frequencies among three time periods were tested by one-way ANOVA, and main activity types were categorized by Steel-Dwass multiple comparison test. D, N, Cr, and Ca indicate diurnal, nocturnal, crepuscular, and cathemeral activity, respectively.

*** indicates *P* < 0.001.

### Seasonal and diel activity patterns

**Fig 4:**
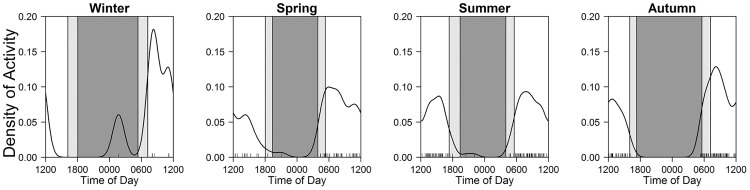
Seasonal patterns of diel activity in Eurasian red squirrels. Black line and bars, dark grey shaded area, and light grey shaded areas indicate kernel density estimates, daily photo events, night-time, and twilight, respectively.

**Fig 5:**
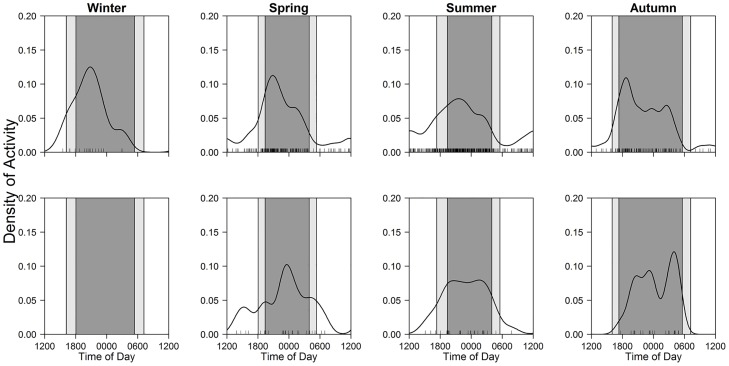
Seasonal patterns of diel activity in raccoon dogs (upper) and raccoons (lower). Black line and bars, dark grey shaded area, and light grey shaded areas indicate kernel density estimates, daily photo events, night-time, and twilight, respectively.

For diurnal and nocturnal mammals, peaks in activity time were largely constant across seasons (i.e. day-time for Eurasian red squirrels and night-time for raccoon dogs and raccoons) (Figs 4 and 5; Table 3). Raccoons, however, were characterized as cathemeral in spring, although a small sample size during this season might have partially affected the results (Fig 5).

**Table 3:** Seasonal patterns of diel activity in diurnal (Eurasian red squirrels) and nocturnal (raccoon dogs and raccoons) mammals.

Eurasian red squirrel					
Season	Twilight	Day-time	Night-time	Category	*P*
Winter	-	0.39 ± 0.16	0.06 ± 0.06	D/N	*
Spring	0.76 ± 0.31	1.95 ± 0.30	0.15 ± 0.11	D	***
Summer	1.22 ± 0.40	4.69 ± 0.51	0.14 ± 0.10	D	***
Autumn	3.13 ± 0.69	4.62 ± 0.60	0.05 ± 0.05	D	***
Raccoon dog					
Winter	0.28 ± 0.20	0.11 ± 0.07	0.76 ± 0.25	Cr/N	*
Spring	4.57 ± 0.87	1.63 ± 0.27	7.70 ± 0.84	N	***
Summer	8.42 ± 1.19	5.37 ± 0.54	14.91 ± 1.38	N	***
Autumn	2.04 ± 0.50	0.66 ± 0.21	4.83 ± 0.55	N	***
Raccoon					
Winter	-	-	-	-	-
Spring	1.02 ± 0.35	0.24 ± 0.10	0.87 ± 0.27	Ca	0.08
Summer	1.49 ± 0.48	0.14 ± 0.08	1.10 ± 0.31	Cr/N	*
Autumn	0.27 ± 0.19	-	0.95 ± 0.22	N	***

These values indicate average photographic frequencies and *SE* per 100 trap-days. Differences in the frequencies among three time periods were tested by one-way ANOVA, and main activity types were categorized by Steel-Dwass multiple comparison test. D, N, Cr, and Ca indicate diurnal, nocturnal, crepuscular, and cathemeral activity, respectively.

*** indicates *P* < 0.001.

* indicates *P* < 0.05.

**Fig 6:**
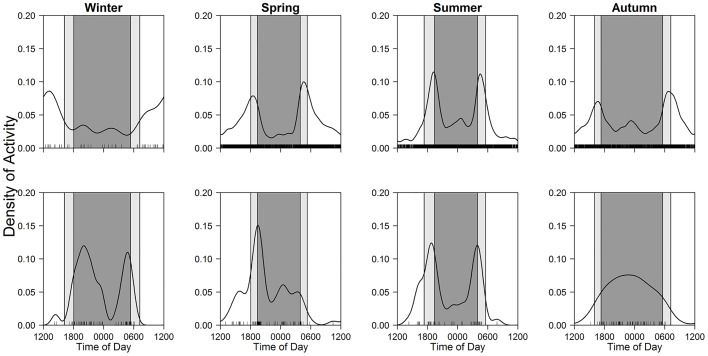
Seasonal patterns of diel activity in sika deer (upper) and mountain hares (lower). Black line and bars, dark grey shaded area, and light grey shaded areas indicate kernel density estimates, daily photo events, night-time, and twilight, respectively.

**Fig 7:**
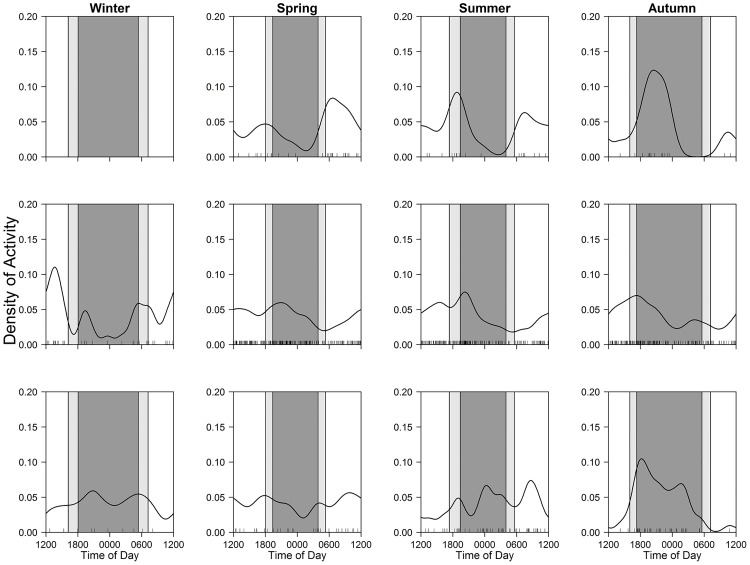
Seasonal patterns of diel activity in brown bears (upper), red foxes (middle), and Japanese martens (lower). Black line and bars, dark grey shaded area, and light grey shaded areas indicate kernel density estimates, daily photo events, night-time, and twilight, respectively.

Some crepuscular and cathemeral mammals shifted peaks across seasons (Figs 6 and 7), though their main activity types were largely constant (Tables 4 and 5). Most notably, sika deer changed peaks from sunrise and sunset during spring–autumn to day-time in winter (Fig 6), but photographic frequencies were not different among three time periods in winter (Table 4). Additionally, mountain hares had peaks at sunrise and sunset during spring–summer, while they showed nocturnal activity during autumn–winter (Table 4; Fig 6).

**Table 4:** Seasonal patterns of diel activity in crepuscular mammals (sika deer and mountain hares).

Sika deer					
Season	Twilight	Day-time	Night-time	Category	*P*
Winter	1.25 ± 0.45	2.35 ± 0.72	0.87 ± 0.27	Ca	0.11
Spring	175.25 ± 10.80	89.25 ± 4.45	41.68 ± 4.31	Cr	***
Summer	241.85 ± 10.93	47.62 ± 2.88	92.63 ± 3.85	Cr	***
Autumn	108.83 ± 7.63	59.82 ± 4.16	45.58 ± 3.38	Cr	***
Mountain hare					
Winter	1.53 ± 0.45	0.15 ± 0.10	2.41 ± 0.37	N	***
Spring	3.81 ± 0.71	0.63 ± 0.19	1.65 ± 0.38	Cr/N	***
Summer	3.80 ± 1.05	0.39 ± 0.14	0.98 ± 0.28	Cr/N	***
Autumn	1.36 ± 0.50	0.06 ± 0.06	2.43 ± 0.37	N	***

These values indicate average photographic frequencies and *SE* per 100 trap-days. Differences in the frequencies among three time periods were tested by one-way ANOVA, and main activity types were categorized by Steel-Dwass multiple comparison test. D, N, Cr, and Ca indicate diurnal, nocturnal, crepuscular, and cathemeral activity, respectively.

*** indicates *P* < 0.001.

**Table 5:** Seasonal patterns of diel activity in cathemeral mammals (brown bears, red foxes, and Japanese martens).

Brown bear					
Season	Twilight	Day-time	Night-time	Category	*P*
Winter	-	-	-	-	-
Spring	0.76 ± 0.35	0.73 ± 0.19	0.22 ± 0.13	Ca	0.21
Summer	0.82 ± 0.33	0.44 ± 0.15	0.19 ± 0.11	Ca	0.13
Autumn	0.14 ± 0.14	0.18 ± 0.10	0.55 ± 0.17	Ca	0.06
Red fox					
Winter	0.83 ± 0.33	1.23 ± 0.30	0.44 ± 0.14	Ca	0.12
Spring	3.81 ± 0.69	4.31 ± 0.50	5.90 ± 0.72	Ca	0.06
Summer	2.31 ± 0.57	3.95 ± 0.43	4.57 ± 0.53	D/N	**
Autumn	3.94 ± 0.72	3.37 ± 0.54	3.45 ± 0.45	Ca	0.76
Japanese marten					
Winter	0.28 ± 0.20	0.22 ± 0.12	0.30 ± 0.12	Ca	0.92
Spring	0.63 ± 0.28	0.82 ± 0.17	0.57 ± 0.19	Ca	0.71
Summer	1.22 ± 0.40	1.06 ± 0.21	1.30 ± 0.31	Ca	0.86
Autumn	0.54 ± 0.27	0.14 ± 0.10	1.45 ± 0.27	N	***

These values indicate average photographic frequencies and *SE* per 100 trap-days. Differences in the frequencies among three time periods were tested by one-way ANOVA, and main activity types were categorized by Steel-Dwass multiple comparison test. D, N, Cr, and Ca indicate diurnal, nocturnal, crepuscular, and cathemeral activity, respectively.

*** indicates *P* < 0.001.

** indicates *P* < 0.01.

For cathemeral species, Japanese martens had no peaks in activity time during winter–summer, but become more nocturnal in autumn (Table 5; Fig 7). Red foxes were categorized as cathemeral during autumn–spring, although their activity peaked during day-time in winter (Table 5; Fig 7). Although the summer activity pattern of this species was not categorized as cathemeral, it was similar to that in spring (cathemeral). For brown bears, they had clear peaks during spring–autumn (spring: day-time, summer: twilight, and autumn: night-time), but we found no significant differences in the frequencies among the three time periods: this may be due to the lack of sample size (Table 5; Fig 7).

### Predator-prey and competitive interactions

For predator-prey interactions, we found no significant influence of red foxes on prey species throughout the year, while the diel niche use of Japanese martens was significantly associated with that of prey species in spring (mountain hares) and autumn (Eurasian red squirrels) (Table 6). On the other hand, we found no evidence of competitive interactions for niche use between raccoons and raccoon dogs or red foxes during spring–autumn (Table 7).

**Table 6:** Results of GLMM for predator-prey interactions between prey species (Eurasian red squirrels or mountain hares) and predator species (red foxes and Japanese martens).

Season	Variables	Eurasian red squirrel				Mountain hare			
		*Estimate*	*SE*	*Z*	*P*	*Estimate*	*SE*	*Z*	*P*
Winter	Intercept	-6.76	0.39	-17.34	***	-4.62	0.16	-28.74	***
	Japanese marten	-4.25	69.36	-0.06	0.95	-6.76	74.12	-0.09	0.93
	Red fox	-5.26	69.34	-0.08	0.94	-7.68	74.06	-0.10	0.92
Spring	Intercept	-4.77	0.17	-28.71	***	-4.73	0.16	-28.74	***
	Japanese marten	-5.51	27.22	-0.20	0.84	1.33	0.67	1.99	*
	Red fox	0.25	0.39	0.66	0.51	-0.62	0.88	-0.70	0.48
Summer	Intercept	-4.15	0.14	-29.62	***	-5.09	0.19	-27.30	***
	Japanese marten	0.96	0.59	1.61	0.11	-5.90	27.19	-0.22	0.83
	Red fox	0.21	0.45	0.47	0.64	0.18	0.65	0.27	0.79
Autumn	Intercept	-4.32	0.15	-29.52	***	-4.63	0.16	-28.76	***
	Japanese marten	1.97	0.46	4.25	***	-6.04	27.05	-0.22	0.82
	Red fox	0.18	0.52	0.34	0.74	-0.12	0.65	-0.19	0.85

The total number of prey species photographed was set as the response variable and the total number of predator species was set as the explanatory variables, using generalized linear mixed model with a Poisson distribution. Each camera site was included as a random intercept.

*** indicates *P* < 0.001.

* indicates *P* < 0.05.

**Table 7:** Results of GLMM for competitive interactions between raccoons and raccoon dogs or red foxes.

Season	Variables	Raccoon			
		*Estimate*	*SE*	*Z*	*P*
Spring	Intercept	-5.54	0.22	-24.72	***
	Raccoon dog	0.64	0.61	1.05	0.29
	Red fox	-7.46	55.45	-0.13	0.89
Summer	Intercept	-5.37	0.21	-25.15	***
	Raccoon dog	0.41	0.43	0.96	0.34
	Red fox	0.41	0.59	0.70	0.48
Autumn	Intercept	-5.66	0.24	-23.75	***
	Raccoon dog	0.48	0.91	0.53	0.60
	Red fox	-6.71	39.00	-0.17	0.86

The total number of raccoons photographed was set as the response variable and the total number of raccoon dogs and red foxes was set as the explanatory variables, using generalized linear mixed model with a Poisson distribution. Each camera site was included as a random intercept.

*** indicates *P* < 0.001.

## Discussion

This intensive camera-trap survey provided one of the most detailed studies of activity patterns in multiple sympatric mammals under natural conditions, and classified seasonal and diel activity patterns into four categories [1]. Surprisingly, no formal classification of the four categories has existed so far. Our ANOVA approach is simple but could be a standard method to evaluate daily activity patterns, although the lack of sample size could lead to misclassification as cathemeral, which might be the case in several species (e.g. brown bears and red foxes) in this study. Although we initially aimed to collect data on sika deer, many sympatric mammals were also recorded. Animal trails were good candidates for this type of camera-trap survey, and the infrared cameras were able to capture mammals of various sizes, ranging from Eurasian red squirrels to brown bears, although mammals smaller than Eurasian red squirrels as well as flying mammals were not monitored. Camera-trap survey is more cost-efficient, in terms of both finance and human effort, than other survey methods, and provides very detailed biological information on seasonal and diel activity patterns throughout the year.

### Seasonal and diel activity patterns of each mammal

Eurasian red squirrels consistently showed a diurnal activity pattern. Red squirrels in Italy also showed diurnal activity, but had a more complex seasonal pattern, with trimodal peaks during summer, bimodal during autumn and unimodal during winter [24]. Similarly, other squirrel species showed diurnal activity with varied peaks. The diel activity patterns of Eurasian ground squirrels (*Spermophilus citellus*) in Hungary showed a peak in the morning [25], whereas those in Bulgaria showed peaks during the afternoon and forenoon during summer and between 13:00 and 15:00 during autumn [26]. In addition, Persian squirrels (*Sciurus anomalus*) showed a peak at midday [27], and the seasonal and diel activity patterns of Mexican fox squirrels (*Sciurus nayaritensis chiricahuae*) in Arizona showed a bimodal distribution (peaks at sunrise and late afternoon) during dry and wet summers and unimodal in day-time during winter [10]. Regardless of the presence of potential predators, such as Japanese martens and red foxes, the diel activity patterns of this species is clearly adapted to diurnal activity.

Raccoon dogs showed nocturnal activity with peaks after sunset throughout the year, although this species was also frequently photographed during the day. Raccoon dogs in Germany, which were introduced from the western Soviet Union in the 1930s–1950s, were more nocturnally active than diurnal during all seasons, and then changed to diurnal activity during pup rearing [28]. In particular, the behavior of the raccoon dogs suggested that males adopted a higher level of diurnal activity to guard the pups. Thus, this species is mainly nocturnal, but may be able to shift its activity to cathemeral when advantageous.

Similar to raccoon dogs, raccoons had night-time peaks throughout the year, although spring activity was categorized as cathemeral. It is generally known that raccoons are nocturnal, but there are few studies of seasonal and diel activity patterns. Pooled data during two sampling periods (summer and autumn) showed this species in east Texas to be active from after sunset to before sunrise [29]. Thus, the diel activity patterns of this species are clearly nocturnal.

Sika deer showed crepuscular activity during spring–autumn, which is consistent with a previous study [19]. We also found that the activity pattern shifted to cathemeral during winter. Most previous studies reported that other deer species are mainly crepuscular (white-tailed deer *Odocoileus virginianus*; [2], elk *Cervus canadensis*; [30], moose *Alces alces gigas*; [31]), whereas white-tailed deer in Michigan were more active during the day-time than at night-time during winter [2]. The day-time activity in winter may be due to reduction of energy consumption, as suggested in adult red deer (*Cervus elaphus*) in Poland (where hunting was prohibited) [32]. Deer species may change seasonal and diel activity patterns.

Mountain hares were more active at twilight during spring–summer, whereas they showed nocturnal activity during autumn–winter. Mountain hares recognize twilight as the onset and cessation of activity [33], displaying clear nocturnal behavior [34], searching for resting sites and food during twilight, and mainly foraging and moving at night [35]. This study confirmed the findings of previous studies: they changed their activity patterns seasonally. A study of mountain hares in Italy suggested that they are mainly nocturnal [33]. Another study of snowshoe hares (*Lepus americanus*) showed that night-time activity was higher during winter than summer [11]. Although hares may change activity patterns, they are clearly inactive during day-time.

Japanese martens mainly displayed cathemeral activity during the study period, except for autumn. Although there was no information on the activity pattern of this species, other marten species showed nocturnal activity. Diel activity patterns of pine martens (*Martes martes*) in Poland was nocturnal (69% of active time) and higher between 18:00 and 06:00 throughout the year [36,37]. Similar patterns were observed for pine martens in Italy photographed between 22:00 and 04:00 from March to June [38]. In Poland, pine martens were killed by lynx (*Lynx lynx*) and red foxes, which were mainly nocturnal, and preyed on yellow-necked mice (*Apodemus flavicollis*), which was active at night [36,37]. Thus, pine martens may synchronize their activity with prey species rather than predator species. In Japan, there is no report on the predators of Japanese martens, whereas prey species are considered to be Eurasian red squirrels and mountain hares, which were diurnal and crepuscular, respectively. Thus, this species may change diel activity patterns depending on the abundance and behavior of the prey species.

Red foxes in our study site were mostly cathemeral throughout the year. Although summer activity was categorized as diurnal or nocturnal, red foxes were photographed throughout the day, and the activity pattern was similar to other seasons. In general, red foxes display nocturnal activity during all seasons in many areas (Japan: [39], Oxford: [40], Bulgaria: [41], Spain: [5]), and culpeo foxes (*Pseudalopex culpaeus*) in Chile are also nocturnal [42]. However, the activity patterns of this species are influenced by availability and activity patterns of prey as well as human disturbance [5,40]. Additionally, this species showed more diurnal foraging behavior in low disturbance environments [43]. Because several prey species (e.g. Eurasian red squirrels and mountain hares) coexist, and the level of human disturbance is low in this study area, this species may adapt from nocturnal to cathemeral.

Brown bears in our study site showed seasonal changes in activity peaks, although the number of photos was limited. This species in Slovenia was also nocturnal during autumn as well as spring, but the yearlings were active throughout the day [44]. Other bear species in different parts of the world showed variability in diel activities: American black bears (*Urusus americanus*) in western Virginia were diurnal during summer and nocturnal during autumn [45], whereas the activity patterns of sun bears (*Helarctos malayanus*) in Borneo were crepuscular throughout the year [46]. Overall, bears species can be active at any time of day.

### Inference of species interactions from camera traps

Recent studies have supported the effectiveness of camera-trap surveys for inferring species interactions, such as predation and competition. However, spatial or temporal comparisons of potentially interacting species are required, and few studies have evaluated possible predator-prey interactions with good spatial replicates [5]. Previous studies reported that predator-prey or competitive interactions were influenced by population density, food availability, temperature, presence of other congeners or food competitors, and predation risk [40,47]. Although there was no support for this data in our study area, prey species (Eurasian red squirrels and mountain hares) may be negatively influenced by the presence of predator species (Japanese martens).

For predator-prey interactions, our data lack a spatial replicate, but show clear temporal changes between potentially interacting species (i.e. Eurasian red squirrels or mountain hares vs. Japanese martens). Additionally, we also found that activity of predator species (Japanese martens and red foxes), which mainly showed cathemeral activity, differed from activity reported in previous studies (nocturnal activity). Thus, the presence of prey species may influence the activity patterns of predator species, though we found no clear evidence to support it. However, differences in local habitat conditions can confound conclusions about species interactions as judged from activity patterns. Therefore, our temporal approach may provide a stronger inference, although effects of other factors influencing diel activity patterns and niche uses should be evaluated to better understand the actual mechanisms involved.

For competitive interactions, it is often cautioned that invasive raccoons can be harmful to native raccoon dogs [48] and red foxes. These species are similar in size, diet, and activity pattern. However, we found no evidence of temporal niche partitioning between these species. This might be because the abundance of raccoons may be too low (much fewer camera-trap events) to affect raccoon dogs and red foxes, or the potentially competitive species interactions may partition diets or habitat use, similar to pumas (*Puma concolor*) and jaguars (*Panthra onca*) [4,49,50]. It is also possible that these species cannot segregate niches and either one of the species could be constantly suffering negative effects.

## Conclusions

We provided detailed seasonal and diel activity patterns of eight sympatric mammals and classified the patterns into four categories by using an intensive camera-trap survey. On the other hand, it was difficult to identify sex and age classes from the photos. In addition, this survey could not distinguish one individual from another, and our results might have represented the activity patterns of a relatively smaller numbers of individuals. Because of these limitations of camera-trap surveys, it would be ideal to combine with other survey tools such as telemetry surveys and direct observations. Particularly, telemetry surveys may provide direct evidence of predator-prey and competitive interactions [51,52]. In addition, we did not evaluate environmental factors affecting diel activity patterns of these mammals, including human disturbances. Thus, a future study is required to clarify various influences on the activity patterns of mammals combining several survey tools and multiple locations.
